# Can the School Fruit and Vegetable Scheme Be an Effective Strategy Leading to Positive Changes in Children’s Eating Behaviours? Polish Evaluation Results

**DOI:** 10.3390/ijerph182312331

**Published:** 2021-11-24

**Authors:** Katarzyna Wolnicka, Anna Małgorzata Taraszewska, Joanna Jaczewska-Schuetz

**Affiliations:** National Institute of Public Health—National Institute of Hygiene—National Research Institute, Department of Nutrition Education, 24 Chocimska Street, 00–791 Warsaw, Poland; ataraszewska@pzh.gov.pl (A.M.T.); jschuetz@pzh.gov.pl (J.J.-S.)

**Keywords:** nutrition, fruit, vegetable, children, school, intervention programme, education

## Abstract

Background: The School Fruit and Vegetable Scheme (SFVS) was developed to form the habit of eating fruit and vegetables (F&V) among children. The survey aimed to identify both the strengths of the scheme and areas that required support and strengthening in the further implementation of school schemes. Methods: The study was conducted from 2012 to 2015 among students of randomly selected 85 primary schools that participated in the programme (intervention group) or did not participate therein (control group). The F&V consumption among the students was evaluated based on the 3 day food record method. Other behaviours were evaluated via frequency and preference questionnaires. Results: Over the three years of implementing SFVS, fruit consumption significantly increased by approximately 30 g/day, i.e., by 18%. In the control group, it increased only by approximately 4%. At the same time, no increase in vegetable consumption was observed. A number of other positive effects of SFVS were also found. These concerned students’ nutritional attitudes and behaviours, such as a further increase in the children’s knowledge on the health aspects of F&V consumption, the levels of their consumption and an increased preference for fruit in general. Conclusions: The results indicate that providing F&V in schools free of charge can be an effective strategy for enhancing F&V consumption among children, in particular by raising the awareness of the health importance of F&V consumption and gradually influencing children’s eating habits, especially when it comes to the habit of fruit consumption. The issue of vegetable consumption is an area for intervention enhancement. There is also a need for further, in-depth analyses, taking into account the impact of potential confounding factors.

## 1. Introduction

The School Fruit and Vegetable Scheme was developed as one of the European Commission’s operations aimed at improving the health and nutrition of children. In Poland, the scheme was carried out from the 2009/2010 to the 2016/2017 school years and was funded from the budget of the EU Common Agricultural Policy. The main principles of the scheme were specified in the Polish National Implementation Strategy for the School Fruit and Vegetable Scheme developed for individual school years.

The School Fruit and Vegetable Scheme aimed to change children’s dietary habits by increasing the consumption of fruit and vegetables in the daily diet at a stage when nutritional habits are shaped. Secondary goals concerned raising the social awareness of proper nutrition, especially when it comes to children, and promoting healthy dietary habits and knowledge on the origin of fruit and vegetables by various educational measures at schools. Schools were obliged to carry out at least two educational activities a year from a specified list of accompanying measures, such as organising thematic competitions and festivals, outings to farms, discussions, healthy food days, cooking classes, running a school newspaper, maintaining a school garden, or having a second breakfast together with tasting fruit and vegetables. The beneficiaries of the School Fruit and Vegetable Scheme in Poland in the evaluated years (2012–2016) were children in Year 1–3 of primary school (6–10 years old). The scheme was very popular—98% of the target group, i.e., more than 1.3 M of children from 11 thousand primary schools, participated in it. As part of the scheme, children received vegetables and fruit free of charge. Children were given fresh fruits (such as apples, pears, strawberries and blueberries), fresh vegetables (such as carrot, sweet pepper, radish, -kohlrabi and cherry tomatoes), as well as fruit, vegetable or mixed juices. The products were selected to include as many fresh fruit and vegetable and regional products of the highest diversity and quality as possible. Fruit, vegetables and juices given away as part of the scheme could not contain added fat, salt, sugar or sweeteners. Each child participating in the scheme received 2–3 portions of fruit and vegetables per week for a total of 20 weeks in a school year. Ready-to-eat portions of fruit and vegetables were prepared and delivered to schools by approved suppliers. The School Fruit and Vegetable Scheme was carried out as part of a cooperation between the sectors of agriculture, public health and education. An authority responsible for implementing the School Fruit and Vegetable Scheme in Poland was Agricultural Market Agency (now, National Support Centre for Agriculture).

The School Fruit and Vegetable Scheme was subject to a regular effectiveness assessment that was to determine whether and how it achieved the set goals. In Poland, the scientific evaluation of the scheme covered the 2011/2012–2015/2016 school years and was carried out by the experts from the Food and Nutrition Institute (now National Institute of Public Health–National Institute of Hygiene–National Research Institute). The aim of the evaluation was to identify the strengths of the scheme and areas requiring support and reinforcement for the further implementation of such school schemes.

From the 2017/2018 school year, the assumptions and goals of the School Fruit and Vegetable Scheme and the School Milk Scheme were combined in the School Scheme.

The results of studies on interventional and educational schemes conducted in the school environment and their effects on nutritional habits and diet quality (including fruit and vegetable consumption) indicate that these can be an effective tool for improving the implementation of nutritional recommendations [1,2,3]. This is confirmed by, for example, effectiveness assessment data concerning fruit and vegetable consumption promotion schemes combined with fruit and vegetables being supplied free of charge at German or Norwegian schools, where their short-term influence on increasing fruit and vegetable consumption or long-term positive influence on fruit consumption (but not vegetables) were demonstrated [2,4,5,6,7].

## 2. Material and Methods

The study was conducted from 2012 to 2016 at randomly selected 85 primary schools that participated or did not participate in the EU School Fruit and Vegetable Scheme among students in Years 1–3 (6–10 years old). The study also involved the children’s parents/legal guardians.

The study was nationwide. It was conducted in five regions of Poland, representing the central, eastern, western, northern and southern areas of the country. Stratified sampling was used, with the school being the basic sampling unit. Before sampling, schools were stratified into urban and rural areas. The study was designed in a way as to make the student sample reflect the structure of population of students attending rural and urban schools in Poland.

At each school, the headmaster chose two classes to participate in a survey. If there was only one class in a school, the next school was chosen. The survey was carried out at primary schools that participated in the School Fruit and Vegetables Scheme (intervention group) and at primary schools that did not participate in the scheme (control group).

In terms of sociodemographic data such as gender of children participating in the study, parental education, net income per family member and number of children in the family, no statistical differences were found between the intervention and control groups. The evaluation study was conducted in the form of a panel study. The evaluation involved several stages from 2012 to 2016.

The study was approved by the Bioethics Committee operating at the Food and Nutrition Institute in Warsaw.

### 2.1. Material

The study involved students from randomly chosen schools who, in 2012, began their school education in Year 1 classes indicated by the headmaster. A total of 3385 students from 85 schools that participated or did not participate in the School Fruit and Vegetable Scheme were invited to take part in the study. The parents of 3113 students gave their informed consent to the participation in the study. In the first year of the study, 2798 students actually took part in it. The same students participated in the study in subsequent school years (study stages) until 2016. Throughout the study, the school sample size decreased, which was due to the joining of schools that, at the beginning of the evaluation, did not participate in the scheme. Nonparticipating schools that joined the scheme during the study did not take part in the further study stages. The student sample size also decreased, which was caused by both the decreasing number of schools included in the study and the absence of students on the survey day, their changed place of residence or a failure to fully complete the survey and the consumption diary. In the third year of the study, an informed consent to the participation in the study was given by the parents of 2631 students in total, with 2251 students actually taking part in the study (Table 1). The final survey return rate in the study amounted to 80.5%.

### 2.2. Research Tool

A research tool for children and parents was an anonymous survey. In addition, children who took part in the study were measured and weighed by a school nurse.

The parent survey contained questions relating to sociological data, open-ended and multiple-choice questions relating to lifestyle, dietary habits with an emphasis on fruit and vegetable consumption, and knowledge on proper nutrition, as well as the child’s fruit and vegetable consumption, dietary habits and lifestyle. The questionnaire included questions relating to the child’s consumption of fresh fruit, vegetables, including salads, other raw vegetables (sliced or whole) and boiled vegetables.

The child survey contained questions related to the knowledge of proper nutrition, dietary habits with an emphasis on fruit and vegetable consumption, lifestyle and dietary preferences, including fruit and vegetables. Children’s knowledge of proper nutrition was measured by an open-ended question “What should you do to live a healthy life?”. Another question was measured by the item “How many portions of fruit and vegetables should you eat?” using a scale ranging from 1 to 7 where, 1 = “should not eat fruit and vegetables at all” and 7 = “5 or more portions per day”, acknowledged as correct knowledge. Children’s dietary preferences were measured by an open-ended question “What do you like to eat?”.

Fruit consumption frequency was determined based on the question related to the fresh fruit consumption frequency. Vegetable consumption frequency was determined based on three questions by adding up the consumption frequency of vegetables in the form of salads, other raw vegetables (sliced or whole) and boiled vegetables (excluding potatoes).

In addition, the students’ dietary habits were assessed using the 3 day food record method. The parents were required to fill in a 3 day food record with the child, where they included products, meals, beverages (along with their weights or home-based measures) consumed by their child during two school days and one weekend day. The parents were trained by the interviewer and additionally received written tips on how to fill in the food record correctly.

The consumption assessment and analysis were carried out using the DIETA 5.0 computer software (National Institute of Public Health-National Institute of Hygiene-National Research Institute, Warsaw, Poland)designed to assess individual and group diet as well as to plan and analyse consumption among tested populations against nutritional standards. It allows for calculating energy and nutritional value of diets as well as the consumption volume of products and dishes. It also ensures standardization of data input regarding products and dishes consumed (order and accuracy of determining the type and composition of dishes), dish recipes, serving size determination (choice of serving sizes suggested in the software), calculation method (identical reduction volumes) and results obtained (ingredient quantity and comparison to standards).

### 2.3. Course of Study

In accordance with the methodology, the study was conducted in two school groups: schools that participated in the scheme (intervention group) and schools that did not participate in the scheme (control group). The survey was conducted near the end of the school year (May/June). The first study stage was an exception, as it took place at the beginning of the school year (October), before the start of actions associated with the School Fruit and Vegetable Scheme.

### 2.4. Statistical Methods

The statistical analysis was conducted using the Statistica 10.0 software (StatSoft Inc., Tulsa, OK, USA 2011). When it comes to the analyses of key study variables, that is fruit and vegetable consumption frequency and consumption expressed in grams, tests of normality (Shapiro-Wilk test) were conducted. Because the distributions of the aforementioned variables significantly deviated from the normal distribution, nonparametric methods were used to verify the hypotheses. For comparisons between the two groups in an independent plan (comparisons between the intervention group and the control group), the Mann–Whitney U test was used; for comparisons in a dependent plan (comparisons between two study stages), the Wilcoxon signed-rank test was used. Basic parameters of descriptive statistics were also calculated: medians, arithmetic means and standard deviations. For comparing proportions in the groups, Pearson’s chi-squared test was used, while for comparisons between measurements, the sign test was used, and numbers and proportions were given. When it comes to variables expressed on ordinal scales, a comparative analysis was additionally performed for means calculated for codes attributed to individual answers. Because this analysis only complemented the previously conducted chi-squared tests and its purpose was to discover potential tendencies to change attitudes, by way of exception, parametric Student’s t-tests were used for independent variables (when comparing groups) and dependent variables (when comparing measurements). The significance level was set at α = 0.05. Results were statistically significant when the calculated test probability was *p* < 0.05.

## 3. Results

### 3.1. Fruit and Vegetable Consumption by Children

**Figure 1:**
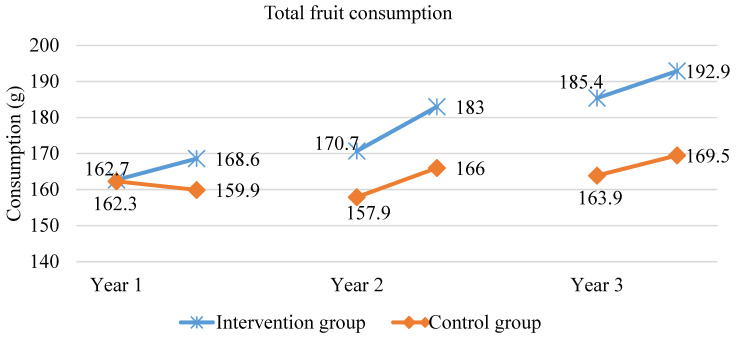
Fruit consumption (on both school and weekend days) during the three years of the study.

**Figure 2:**
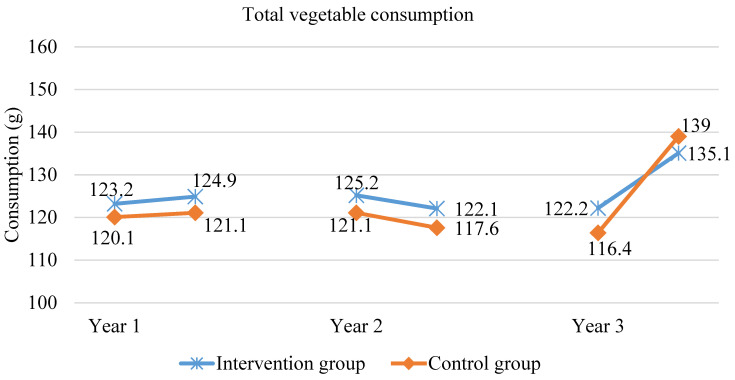
Vegetable consumption (on both school and weekend days) during the three years of the study.

**Figure 3:**
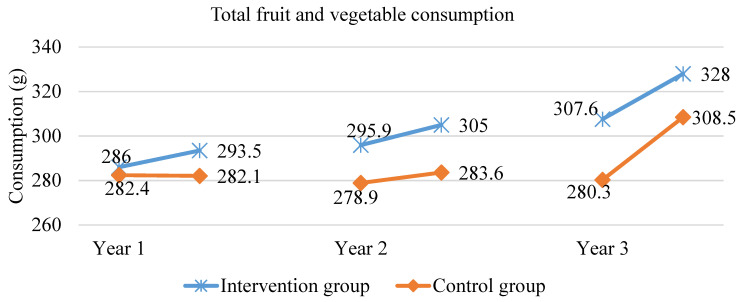
Total fruit and vegetable consumption (on both school and weekend days) during the three years of the study.

After one year in the scheme, the amount of consumed fruit (*p* < 0.0009), vegetables (*p* < 0.027) and fruit and vegetables together (*p* < 0.0003) significantly increased among the children who were included in the intervention. The results related to fruit and vegetable consumption (on school and weekend days) are shown in Figure 1, Figure 2 and Figure 3. At the same time, the control group recorded a lower amount of fruit consumed (*p* < 0.043), while the amount of vegetables did not significantly change. After one year in the scheme, the intervention group consumed 11% more fruit than the control group and 9% more fruit and vegetables together. Two years in the scheme, on school and weekend days, in the intervention group, the total fruit and vegetable consumption significantly increased by 9 g (*p* = 0.004), which was mainly due to increased fruit consumption (*p* < 0.00001 for fruit alone). In the control group, no statistically significant increase in the total fruit and vegetable consumption was recorded, but there was a statistically significant increase in fruit consumption (*p* = 0.01), although lower than in the intervention group. At the end of the second year of intervention, the total fruit and vegetable consumption in the intervention group was still higher—the difference was 21 g, which constitutes a 7% difference (*p* = 0.0008). On both school and weekend days, in the group included in the scheme, the consumption of fruit and vegetables together significantly rose (by 20.4 g; *p* < 0.000) after three years, which was due to the increased consumption of both fruit and vegetables. In the control group, there also was a statistically significant increase in the consumption of fruit and vegetables together, which was mainly associated with the increase in vegetable consumption. However, at the end of the third year of the intervention, in the group included in the scheme, the total fruit and vegetable consumption on school and weekend days together was still significantly higher than in the control group (which was attributed to higher fruit consumption in the intervention group than in the control group; 19.5 g difference, *p* = 0.018).

Throughout the three years of the School Fruit and Vegetable Scheme, fruit consumption increased by 30 g/day, i.e., 18%, while in the control group by 4%. Vegetable consumption remained at a similar level for the first two years of the scheme, while in the third year, it rose significantly in both groups (no statistically significant differences between the groups at the end of the scheme).

### 3.2. Children’s Knowledge on Proper Nutrition

**Figure 4:**
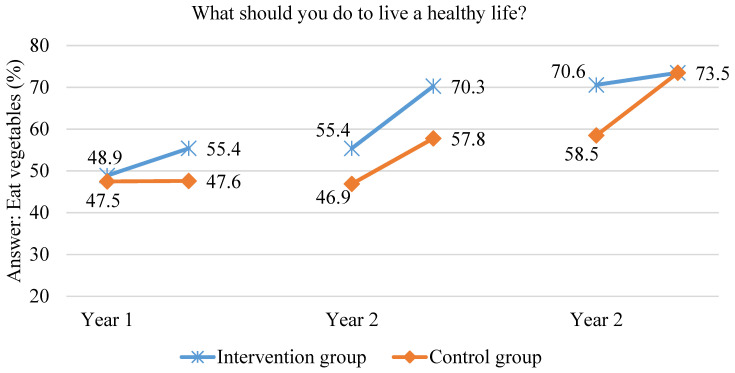
Proportion of children indicating vegetable consumption as a health-supporting behaviour during the three years of the study.

**Figure 5:**
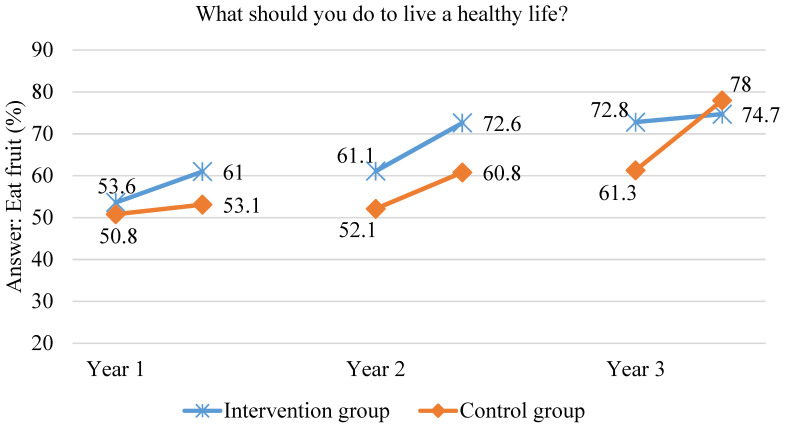
Proportion of children indicating fruit consumption as a health-supporting behaviour during the three years of the study.

There was a significant rise in children’s awareness in link between fruit and vegetable consumption and health in the first year of the scheme. After one year in the scheme, to the open-ended question “What should you do to live a healthy life?” in the intervention group, significantly more children answered “eat vegetables” and “eat fruit” but also “be more physically active” and “do not eat sweets”. In the second year of the scheme, the children’s awareness that eating fruit and vegetables is important for their health was maintained. In the intervention group, the proportion of children indicating “vegetable consumption” increased by 14.9%, while in the control group by 10.9%. At the end of the second year of the scheme, in the intervention group, children significantly more often answered “eat vegetables” compared to the control group children (70.3% vs. 57.8%, respectively, *p* < 0.00001). In addition, the link between fruit consumption and health was pointed by significantly more children in the intervention group—72.6% compared to 60.8% of children from the control group (*p* < 0.000001). In the third year of the study, in the intervention group, the percentage of children arguing that one needs to eat fruit and vegetables in order to be healthy did not significantly change relative to the previous year. In the control group, however, the number of children arguing that one needs to eat fruit and vegetables in order to be healthy increased to the level of the intervention group. At the end of the third year, the results achieved did not significantly differ between the intervention group and the control group. The data are shown in Figure 4 and Figure 5. In both groups, after three years in the scheme, approximately three out of four children indicated that one needs to eat fruit and vegetables in order to be healthy. The level of knowledge on this matter in the control group matched the higher level of knowledge among the intervention group children achieved in the previous two years.

**Figure 6:**
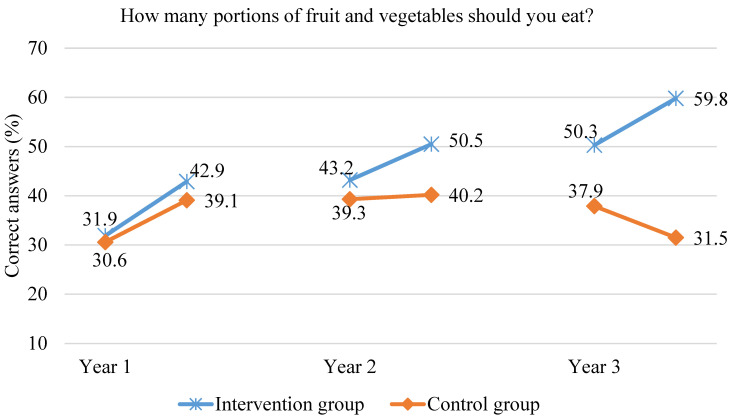
The proportion of children correctly answering the question “How many portions of fruit and vegetables should you eat?” during the three years of the study.

After the first year, there was a significant increase in the knowledge of the children participating in the scheme on the recommended daily portion of fruit and vegetables. In the second year of the scheme, the proportion of correct answers related to the recommended daily portion of fruit and vegetables increased again (by 7.3%, *p* = 0.001), while in the control group, this knowledge did not change. At the end of the second year of the study, the intervention group children answered correctly more often than the control group children—50.5% and 40.2% of correct answers, respectively (*p* < 0.000). After the third year of the scheme, in the intervention group, there was a further increase (by 9.5%) in the proportion of children having knowledge of the recommended vegetable and fruit portions. At the same time, a significant drop in the number of children correctly answering the question “How many portions of fruit and vegetables should you eat?” in the control group was observed. At the end of the third year of the scheme, significantly more children in the intervention group had correct knowledge on the recommended daily portion of fruit and vegetables (59.8% vs. 31.5%; *p* < 0.000). The data are shown in Figure 6.

### 3.3. Children’s Dietary Preferences

**Figure 7:**
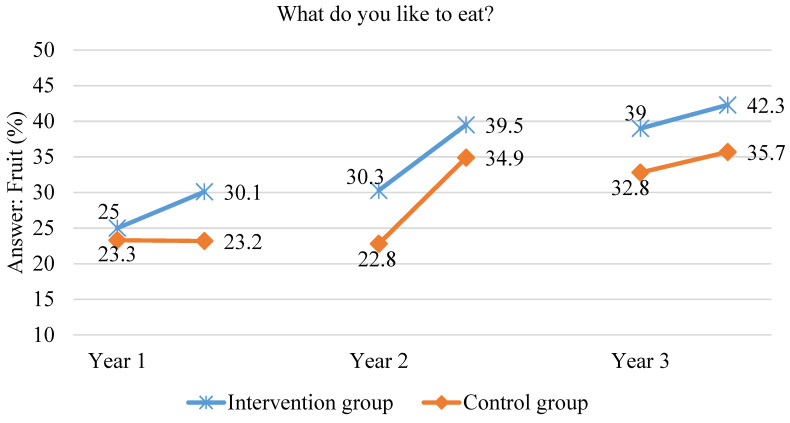
The proportion of children indicating fruit as products they like to eat during the three years of the study.

**Figure 8:**
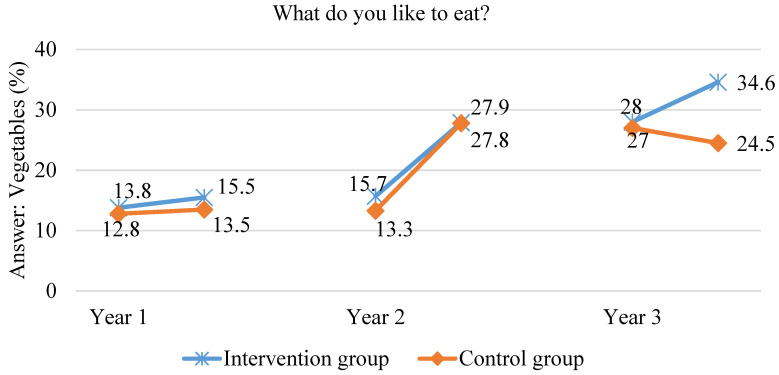
The proportion of children indicating vegetables as products they like to eat during the three years of the study.

After one year in the scheme, to the question “What do you like to eat”, “fruit” was the answer given more frequently in the intervention group than in the control group (30.1% vs. 23.3%; *p* = 0.0001). Only 15.5% of the students from the intervention group and 13.5% of the students from the control group indicated vegetables after one year in the scheme, but the differences were not significant. After two years in the scheme, children in the intervention group also answered “fruit” to the aforementioned question more often than in the control group (39.5% vs. 34.9%, respectively, *p* = 0.03). In both groups, fewer children indicated vegetables as products they liked, as opposed to fruit. After three years in the scheme, the number of children indicating vegetables as products they liked was significantly higher in the intervention group than in the control group (34.6% vs. 24.5%; *p* < 0.000). What is more, in the intervention group, the number of children who liked fruit was significantly higher (42.3% vs. 35.7%; *p* = 0.007). The data are shown in Figure 7 and Figure 8.

## 4. Discussion

Health-supporting dietary habits developed in childhood translate into children’s health and correlate with adult health, reducing the risk of numerous noninfectious diseases [8,9,10].

One of the important aspects of a health-supporting nutritional model is the regular consumption of fruit and vegetables. According to the recommendations by World Health Organisation (WHO), one should consume at least 400 g of fruit and vegetables with a frequency of at least five portions a day [11]. However, studies indicate that achieving this minimum is problematic. The results of the IDEFICS study suggest that only 8.8% children meet the recommendations on fruit and vegetable consumption [12]. Children aged 6–11 consume, on average, from two to three portions of fruit and vegetables per day, as suggested by studies carried out in Europe, Australia and the United States [13].

Studies indicate that the consumption of fruit and vegetables by children and adolescents in Poland is also unsatisfactory. According to the COSI study (2016), only 23.8% of 8 year-olds consumed vegetables every day, while 63.4% consumed them several times a week. When it comes to fruit, 35.7% children consumed them every day, while 59% children had fruit on several days in a week [14]. The HBSC study (2018) conducted among a group of adolescents aged 11–15 demonstrated that 38.2% of teenagers consumed fruit and 34.2% vegetables with recommended frequency. Daily fruit consumption became increasingly rarer with age in both sexes. The lowest proportion of adolescents consuming vegetables at least once a day was recorded for 13 year-olds, both boys and girls. Compared to the results of the HBSC study of 2014, the proportion of adolescents who had vegetables every day rose by 4.9%, while fruit rose by 4.4% [15,16]. Therefore, there is still a need to conduct various campaigns to popularise health-supporting habits, including an increase in fruit and vegetable consumption among children and adolescents. One of such campaigns was the EU educational and interventional School Fruit and Vegetable Scheme, implemented in Poland since 2009.

An extremely important aspect of conducting such schemes is their evaluation in order to assess the effectiveness of actions taken and determine areas that require corrections. The analysis of data from the evaluation of the Polish edition of the School Fruit and Vegetable Scheme indicated that, over the three years of the scheme, fruit consumption significantly increased by approximately 30 g per day, i.e., 18%. Although this amount is not significant, it indicates an upward trend in the consumption of food products, which are so crucial for the proper functioning of the young organism. At the same time, in the control group, it only rose by approximately 4%. Vegetable consumption remained at a similar level for the first two years of the scheme, while in the third year, it rose significantly in both groups (no statistically significant differences between the groups at the end of the scheme). The results showed positive effects of the scheme mostly on fruit consumption. Studies by other authors also confirmed a higher preference for fruit compared to vegetables [17]. The reason for this preference can be fruit’s sweet flavour as opposed to vegetables. The literature’s data indicate that the preference for sweet and salty flavours is the highest in the early childhood and decreases slightly with age [18,19].

Despite higher fruit consumption observed as a result of the scheme, the low total fruit and vegetable consumption as compared to the WHO recommendations is still a concern, as is the minimal effect of the scheme on vegetable consumption. The small effect of the School Fruit and Vegetable Scheme on vegetable consumption can be caused by the fact that, one portion of fruit and vegetables made available twice a week consisted of less vegetables (60 g) than fruit (100–150 g). Additionally, it needs to be noted that juices were distributed four times in 10 weeks.

Previous studies related to the effectiveness of schemes aimed at increasing fruit and vegetable consumption among children showed that it is easier to boost fruit consumption than vegetable consumption due to fruit’s sweet flavour being better tolerated by children [4,20].

One of these studies assessed the effectiveness of the School Fruit and Vegetable Scheme and the influence of the frequency of providing fruit and vegetable portions on changing children’s habits. Supplying fruit for three days, but also for two days, in a school week caused an increase in fruit and vegetable consumption among children. It was observed that children who attended an after-school club derived greater benefits from the supply of fruit to school, which the authors attributed to the fact that the fruit and vegetable that were left in the morning were given to children participating in an after-school club. Thus, students who spent a whole day at school had more opportunities to access fruit and vegetables. The authors conclude that the EU School Fruit and Vegetable Scheme is generally a useful tool aimed at increasing the consumption of fruit and vegetables by primary school students [21].

In the School Fruit and Vegetable Scheme, the practical aspect of free distribution of fruit and vegetables among children at school is important. The authors of a study evaluating the effectiveness of the *5 a day for KIDS* scheme concluded that 135 min educational classes at school did not seem to increase fruit and vegetable consumption among children. According to the researchers, the scheme’s effectiveness can be improved, e.g., by increasing the involvement of parents and/or supplying free fruit/vegetables every day [22]. In another study, it was demonstrated that the School Fruit and Vegetable Scheme increased fruit and vegetable consumption in a group that received fruit free of charge. The effect was evident after three months of distribution. After seven months, the effect remained significant, although it decreased and returned to the baseline level in the second year, when the students were no longer included in the scheme. The scheme brought a short-term effect of increased fruit and vegetable consumption. The authors argue that further studies on the influence of factors that bring long-term effects of increased fruit and vegetable consumption among children are necessary [23].

A systematic review of 10 interventions increased fruit and vegetables intake in primary school children, resulting in a significant effect on fruit and vegetable consumption, ranging from 0.3 to 0.99 portions a day [24].

The School Fruit and Vegetable Scheme is also an educational scheme consisting of accompanying measures aimed at increasing children’s and parents’ knowledge, as well as reinforcing health-supporting behaviours among students included in the scheme.

As a result of the programme, there was an increase in children’s awareness of the importance of eating fruit and vegetables for their health, an increase in knowledge about the recommended number of daily portions of fruit and vegetables and an increase in preference for this group of products.

It can be assumed that these positive observations relating to knowledge and preferences were affected by the educational measures conducted as part of the scheme. The School Fruit and Vegetable Scheme, which relied both on supplying fruit and vegetables and on conducting accompanying educational measures, constitutes good practice that contributes to increasing children’s knowledge on healthy nutrition. Children who did not participate in the scheme showed poorer knowledge on the recommended number of fruit and vegetable portions and more rarely answered that they liked to eat vegetables. The evaluation results indicate that the children’s knowledge and awareness of the health impact of fruit and vegetables and recommendations on their consumption systematically rose.

However, it needs to be noted that, after three years of participation in the scheme, only slightly more than half of the intervention group students were able to tell the correct recommended number of fruit and vegetable portions. This leads to the conclusion that information passed in the form of accompanying educational measures may be insufficient, incoherent or delivered in a manner that is not attractive enough. Accompanying measures as an integral part of the scheme should, then, be reinforced and continued at various levels of student’s school education. Educational measures that accompany the School Fruit and Vegetable Scheme should, first and foremost, be supported by a package of unified and systematised educational materials to be used by the teachers of all the schools participating in the scheme, including lesson scenarios, teaching aids, both for working with the students and their parents, which would help the schools achieve set goals in an attractive and unified way.

It has been proven that the most effective educational programmes on correct nutrition and physical activity are based in the school environment. The school environment, including students, teachers and other school employees, as well as parents or even the local community, provides numerous opportunities to spread knowledge in the form of various measures and activities reinforcing health-supporting behaviours, including higher fruit and vegetable consumption. In light of the increasing issue of overweight and obesity among children and adolescents, it is necessary to organise health-promoting educational measures covering the entire school environment [6,7,8].

In accordance with expert recommendations, prevention schemes aimed at increasing the knowledge and shaping correct nutritional attitudes and habits require far-reaching measures. Single actions or interventions do not bring expected results and should be a part of long-term programmes [8,25].

A significant factor that affects children’s fruit and vegetable consumption are parents’ dietary habits. Numerous studies suggest that higher fruit and vegetable consumption among school-aged children depend on such familial factors as the parents’ fruit and vegetable consumption, encouraging the child to eat fruit and vegetables, giving the child fruit and vegetables to school, the availability of fruit and vegetables at home and the parents’ knowledge on the recommended fruit and vegetable intake. The results indicate the great importance of educational measures targeted at parents, which are needed to increase fruit and vegetable consumption among children [26,27,28,29]. Parents’ dietary habits and their health-supporting behaviours play an extremely significant role in shaping the children’s preferences for fruit and vegetables, which means that working with parents on an ongoing basis, with an emphasis on health-promoting education, should be an inherent part of the scheme’s accompanying measures. Schools should have tools at their disposal for conducting systematised and planned educational measures targeted at parents. Working with parents gives the chance to create a unified educational environment and, as a result, a higher effectiveness of measures aimed at increasing fruit and vegetable consumption.

The choice of fruit and vegetables shared as part of the scheme should be diverse. Expanding the range of products offered to children at school and thus making it more attractive, provided that it spans for a longer period, might contribute to increasing the effectiveness of measures conducted.

Headmasters should enforce the quality of products delivered by suppliers because reduced quality, even if it affects only some product batches, may influence the food characteristics that children find important, such as sweetness, crispiness or juiciness, and constitute a critical point affecting the consumption among children and the scheme’s effectiveness.

According to the authors, a strength of the study is that it compares changes not only between the different stages of the study (years) but also between the intervention and control groups. There were some limitations of the study. In the present study, all data were self-reported by parents with children and thereby may be limited by their comprehension and memory. Another limitation was that the sample size at the last year of the study was lower than planned for the control group, which was due to the joining of schools which, at the beginning of the evaluation, did not participate in the scheme. A limitation related to data analysis might be the fact that observed differences between study groups might not be due only to the intervention but also to the changes in individual or school-level characteristics. This is particularly important in case, as many schools were dropped from the control group in later years of the intervention. However, in terms of sociodemographic data such as gender of children participating in the study, parental education, net income per family member and the number of children in the family, no statistical differences were found between the intervention and control groups in every year/stage of survey. In addition, the analyses were conducted each year, between years, in the form of a panel study in the same group of children.

## 5. Conclusions

Supplying portions of fruit and vegetables free of charge at school can be an effective strategy leading to increased fruit and vegetable consumption among children. Participation in a scheme whereby fruit and vegetables are supplied along with conducting accompanying measures raises the awareness of the health importance of eating fruit and vegetables and the gradual effects on children’s dietary habits, especially when it comes to the habit of eating fruit. The issue of vegetable consumption is an area for intervention enhancement. There is also the need for further, in-depth analyses, taking into account the impact of potential confounding factors.

## Figures and Tables

**Table 1:** Changes in the sample size of children taking part in the evaluation study in the years 2012–2015.

Year of Study	Total Number of Children Invited to the Study	Total Number of Children Taking Part in the Study	Number of Schools Participating in the Study		Number of Children Participating in the Study	
			Included in the Scheme	Not Included in the Scheme	Included in the Scheme	Not Included in the Scheme
2012	3385	2798	41	44	1518	1280
2013	3333	2773	41	44	1500	1273
2014	3003	2075	41	36	1269	806
2015	2631	2251	41	25	1463	788
